# Digitale Transformation in den Haushalten älterer Menschen

**DOI:** 10.1007/s00391-021-01897-5

**Published:** 2021-04-28

**Authors:** Alexander Seifert

**Affiliations:** 1 Zentrum für Gerontologie, Universität Zürich, Pestalozzistraße 24, 8032 Zürich, Schweiz; 2 Fachhochschule Nordwestschweiz, Hochschule für Soziale Arbeit, Olten, Schweiz

**Keywords:** Smartphone, Tablet, Technik, IKT, Techniknutzung, Schweiz, Smartphone, Tablet, Technology, ICT, Technology use, Switzerland

## Abstract

**Hintergrund:**

Nicht nur jüngere, sondern zunehmend auch ältere Menschen leben heute in einer Welt, in der digitale Alltagstechnologien ihren Alltag maßgeblich begleiten. Aber wie hat sich diese Techniknutzung in den letzten 10 Jahren verändert, und inwieweit hat sich die Einstellung gegenüber dieser Technik verändert? Um diese Fragen zu beantworten, wurden 3 querschnittliche Bevölkerungsbefragungen miteinander verglichen.

**Material und Methoden:**

Das Datenmaterial stammt aus 3 Schweizer Befragungen (2009: *n* = 1105, 2014: *n* = 1037, 2019: *n* = 1130) von Personen ab 65 Jahren. Diese Befragungen erfolgten jeweils als standardisiertes telefonisches Interview und wurden mit einer optionalen schriftlichen Befragung kombiniert.

**Ergebnisse:**

Sowohl die Nutzung des Internets als auch die von mobilen Endgeräten (Smartphone, Tablet) ist von 2009 zu 2019 angestiegen. So nutzten 2009 37,8 % der Befragten das Internet, 2019 waren es bereits 74,2 %. Dennoch ist weiterhin zu erkennen, dass v. a. Personen ab 80 Jahren diese Technologien seltener nutzen. Auch wenn 2019 bereits mehr unterschiedliche Internetanwendungen genutzt wurden als noch 2009, so wurden jedoch ähnliche Gründe für die Nichtnutzung des Internets angegeben. Zu den Hauptgründen zählen Sicherheitsbedenken und Angaben, nach denen die Nutzung des Internets zu kompliziert bzw. das Erlernen des Umgangs mit dem Internet überhaupt zu hoch sei. Auch die Einstellungen zur Technik und die Faktoren, die für eine Internetnutzung sprechen, haben sich nur wenig verändert.

**Schlussfolgerung:**

Auch wenn die digitale Transformation voranschreitet, gibt es bei der Techniknutzung weiterhin Ungleichheiten. Auch wenn sich diese mehr und mehr nivellieren, so werden neue Technologien in den kommenden Jahren neue Ungleichheiten schaffen.

**Zusatzmaterial online:**

Zusätzliche Informationen sind in der Online-Version dieses Artikels (10.1007/s00391-021-01897-5) enthalten.

## Abstract (en)

**Background:**

Digital technologies play an important role in people’s everyday lives and this applies not just to younger people but increasingly to older people. The study examined how the use of and attitudes towards such technology have changed over the last 10 years through a comparison of 3 cross-sectional population surveys.

**Material and methods:**

The data came from three Swiss surveys (2009: *n* = 1105; 2014: *n* = 1037; 2019: *n* = 1130) of people aged 65 years and over, each of which was conducted as a standardized telephone interview and combined with an optional written survey.

**Results:**

The use of both the internet and mobile devices (smartphones, tablets) increased from 2009 to 2019: in 2009, 37.8% of the respondents used the internet, rising to 74.2% in 2019; however, people over 80 years old used these technologies less frequently. Although more and different internet resources were being used in 2019 than in 2009, the reasons given for not using the internet were similar and included security concerns, claims that using the internet is too complicated and the belief that learning to use it is too difficult. There has also been little change in the attitudes toward technology and the factors explaining internet use.

**Conclusion:**

Even though the digital transformation is progressing, inequalities in the use of technology remain. Even when these are levelling out, new technologies will likely create new inequalities.

Unser Alltag wird zunehmend digital, ob beruflich oder privat – die aktuelle Coronapandemie hat dies noch einmal verdeutlicht. Doch wie sieht die digitale Transformation in den Haushalten der Personen ab 65 Jahren aus, und wie hat sich dort die Nutzung seit 2009 verändert? Diese Kernfragen waren leitend für die hier näher vorzustellende Vergleichsanalyse auf Grundlage von 3 Bevölkerungsbefragungen aus den Jahren 2009, 2014 und 2019.

## Hintergrund und Fragestellung

Gerade in der aktuellen COVID-19-Pandemie zeigte sich die Digitalisierung unseres Alltags noch einmal deutlich, im Sinne einer zunehmenden digitalen Kommunikation (z. B. durch Homeoffice). Diese zunehmende digitale Transformation zeigt sich u. a. auch im Nutzungsanstieg moderner Informations- und Kommunikationstechnologien (IKT) wie dem Internet oder dem Smartphone. Dennoch ist aus der gerontologischen Forschung bekannt, dass auch heute noch Formen der digitalen Spaltung zwischen jüngeren (unter 65 Jahre) und älteren Altersgruppen (über 65 Jahre) bestehen [1, 3]. So zeigen Studien aus Europa, dass z. B. nur 53 % der Personen ab 50 Jahren das Internet nutzen [5].

Jüngere Menschen leben heute ganz selbstverständlich in einer digitalisierten Lebenswelt. Anders ist dies bei älteren Personen, die mit diesen Technologien nicht groß geworden sind und somit weniger Berührungspunkte damit haben [12]. Auch wenn moderne Technologien wie z. B. eine Erinnerungshilfe auf dem Smartphone eine Hilfe im Alter darstellen können [7], zeigen Studien auch, dass älteren Menschen z. T. die nötigen Technikkompetenzen fehlen oder dass sie keinen direkten Vorteil in der Nutzung sehen, da sie bisher von den „klassischen Wege“ wie z. B. direkten Kontakten oder dem Gang zum Bahnschalter im Alltag Gebrauch machen [16–18]. Im Alter kann sich zudem die körperliche und kognitive Funktionsfähigkeit verändern. Zudem können soziale oder finanzielle Ressourcen eingeschränkt sein oder Ängste gegenüber der Nutzung von Technik bestehen, was den Zugang zu dieser wiederum erschwert [2]. Die aktuelle COVID-19-Pandemie hat den digitalen Graben zwischen Jung und Alt noch einmal deutlicher gemacht, da Personen ohne technische Kompetenzen und Mittel fehlende direkte soziale Kontakte nicht durch technische Lösungen kompensieren können [16].

Unklar bleibt jedoch, wie weit die digitale Transformation dennoch auch in der Bevölkerungsgruppe der ab 65-Jährigen vorangeschritten ist, und inwieweit sich diese digitale Transformation der Alltagsanwendungen auf deren Einstellung zu dieser Technik generell verändert hat, bzw. ob sich auch die begründeten Faktoren einer Internetnutzung über die Jahre verändert haben. Internationale Studien [1–3, 5] liefern erste Hinweise darauf, dass einerseits beeinflussende Faktoren z. B. der Internetnutzung sich über die Jahre angepasst haben. Antworten auf diese Fragen sollen die Nutzungsprofile von Alltagstechnologien wie dem Internet, Smartphone oder Tablet geben. Hierbei soll anhand von deskriptiven Vergleichen bestehender Untersuchungen aus der Schweiz einerseits der zeitliche Wandel in der Techniknutzung, Technikinteresse, Anwendungsvielfalt und möglichen Hürden der Techniknutzung gegenübergestellt werden.

Diese Fragen waren Ausgangspunkt vorliegender Vergleichsstudie. Grundlage des Vergleichs liefern 3 Schweizer Querschnittserhebungen aus den Jahren 2009, 2014 und 2019; welche hier erstmals deskriptiv verglichen werden sollen, um die oben genannten zeitlichen Veränderungen darzustellen. Es soll hierbei eruiert werden: (a) wie sich die Einstellungen zur Technik und zum Internet in der Zeit von 2009 bis 2019 verändert haben, (b) wie sich die Internetnutzung und die Nutzung von Alltagstechnologien (alltägliche technische Geräte im Haushalt z. B. Radio, Mobiltelefon, Tablet) über die Jahre verändert haben, (c) welche Gründe der Nichtnutzung des Internets benannt werden, (d) welche Anwendungen im Internet über die Jahre bevorzugt werden, und (e) welche Einflussfaktoren für die Internetnutzung sich in den jeweiligen Untersuchungen ergeben.

## Studiendesign und Untersuchungsmethoden

### Daten

Grundlage für die Auswertung waren 3 repräsentative – im Querschnitt in der Schweiz erhobene – Bevölkerungsbefragungen von in Privathaushalten lebenden Personen ab 65 Jahren aus den Jahren 2009 [10], 2014 [17] und 2019 [14]. Die Stichprobe im Jahr 2009 beinhaltet *n* = 1105 Personen im Alter zwischen 65 und 97 Jahren (*M* = 73,8; *SD* = 7,11), die Stichprobe aus dem Jahr 2014 beinhaltet *n* = 1037 Personen im Alter zwischen 65 und 100 Jahren (*M* = 74,5; *SD* = 6,96) und die Stichprobe aus dem Jahr 2019 beinhaltet *n* = 1130 Personen im Alter zwischen 65 und 101 Jahren (*M* = 74,1; *SD* = 6,69). Die Charakteristik der 3 Stichproben kann Tab. 1 entnommen werden. Alle 3 Befragungen fanden jeweils in den Monaten August bis Oktober und in allen Sprachregionen der Schweiz statt. Die Interviews mit standardisierten Fragen wurden telefonisch („computer assisted telephone interview“, CATI) geführt und durch eine schriftliche Erhebung von Haushalten ohne Telefonanschluss ergänzt. Die Stichproben wurden mittels einer Wahrscheinlichkeitsauswahl aus der Grundgesamtheit der ständigen Wohnbevölkerung der Schweiz ab 65 Jahren bestimmt.

**Table 1:** 

		Prozente		
Merkmal	Kategorien	2009 (*n* = 1105)	2014 (*n* = 1037)	2019 (*n* = 1130)
Geschlecht	Frauen	54,7	52,7	51,0
	Männer	45,3	47,3	49,0
Altersgruppen	65–79 Jahre	77,2	76,7	78,7
	80+	22,8	23,3	21,3
Wohnsituation	Allein lebend	35,4	36,1	30,9
	Nicht allein lebend	64,6	63,9	69,1
Bildungsstand	Bis Sekundarstufe I	21,8	18,2	13,0
	Sekundarstufe II	57,8	57,3	53,4
	Tertiärstufe	20,3	24,5	33,6
Haushaltseinkommen	Bis CHF 4.000	40,0	38,1	29,7
	CHF 4001 bis 8000	45,1	44,1	48,7
	Über CHF 8.000	14,9	17,8	21,6
Subjektive Gesundheit	Sehr gut	25,9	46,9	44,4
	Eher gut	45,8	30,8	25,5
	Teils, teils	21,8	14,4	19,5
	Eher schlecht	5,4	3,9	5,0
	Sehr schlecht	1,1	3,9	5,6

Abgebildet sind die Häufigkeitsverteilungen der ungewichteten Stichproben

### Messinstrumente

Es wurden folgende Variablen zum deskriptiven Vergleich genutzt (Tab. 2):

1. Technikinteresse ([10]; 2019: M = 3,50 (SD = 0,96), Cronbachs α = 0,63);
2. Einstellungen zum Internet ([10]; 2019: M = 3,35 (SD = 1,16), Cronbachs α = 0,75);
3. Besitz von IKT (Skala: 1 = ja, 0 = nein, [10]);
4. Internetnutzung („Haben Sie das Internet in den letzten 6 Monaten genutzt?“, Skala :1 = ja, 0 = nein, [10]);
5. Gründe der Nichtnutzung des Internets (Skala: 1 = Grund, 0 = kein Grund, [10]);
6. genutzte Internetanwendungen (Skala: 1 = ja, nutze ich, 0 = nein, nutze ich nicht, [10]);
7. Kontrollvariablen (Altersgruppen, Geschlecht, Bildung, Haushaltseinkommen, alleinlebend, subjektive Gesundheit).

Mittelwerte sowie prozentuale Verteilungen der Variablen sind Tab. 1**bzw.** 2 zu entnehmen.

**Table 2:** 

Aspekte	2009	2014	2019	2009	2014	2019
	Mittelwert (SD)			Mittelwerte nach Altersgruppen (65–79/80+)		
*Technikinteresse*^*1*^						
(1.1) Der technische Fortschritt muss immer weitergehen	3,85 (1,275)	3,82 (1,249)	3,71 (1,204)	3,86/3,83	3,86/3,71	3,67/3,78
(1.2) Ohne technische Geräte könnte ich mir mein Leben nicht mehr vorstellen	3,75 (1,390)	3,61 (1,427)	3,59 (1,356)	3,79/3,66	3,65/3,49	3,65/3,43*
(1.3) Ich interessiere mich sehr für neue technische Dinge	2,97 (1,418)	2,96 (1,395)	3,07 (1,270)	3.09/2,66***	3,07/2,66***	3,18/2,80***
*Einstellung zum Internet*^*2*^						
(2.1) Das Internet erleichtert den Kontakt zu anderen Menschen	3,18 (1,551)	3,21 (1,550)	3,28 (1,487)	3,24/3,02	3,32/2,91***	3,33/3,15
(2.2) Das Internet erspart viel Lauferei	3,32 (1,488)	3,47 (1,512)	3,35 (1,433)	3,46/2,87***	3,57/3,17**	3,44 /3,10**
(2.3) Das Internet ist anregend und faszinierend	3,21 (1,533)	3,41 (1,459)	3,23 (1,409)	3,34/2,80***	3,50/3,13**	3,37/2,87***
*IKT-Besitz/-Nutzung*	**2009**	**2014**	**2019**	**2009**	**2014**	**2019**
	**Prozente Besitz (davon tägliche Nutzung)**			**Prozente nach Altersgruppen** (65–79/80+)		
Fernseher	96,7 (89,0)	95,8 (92,6)	95,3 (87,4)	96,6/96.8	96,0/95,4	95,4/94,7
Radio	94,4 (79,9)	94,7 (78,9)	89,6 (75,9)	95,5/91.8	96,3/90,5***	92,0/83,4***
Festnetztelefon	96,9 (32,3)	71,2 (61,6)	84,5 (51,5)	96.6/97,7	95,2/96,5	81,1/94,3***
Klassisches Mobiltelefon	76,7 (16,6)	71,2 (30,8)	47,0 (43,0)	87,5/48,8***	74,8/62,3***	41,0/61,0***
Smartphone	–	29,6 (62,7)	63,7 (78,1)	–	37,3/10,1***	75,2/34,6***
Tablet	–	23,9 (51,1)	40,0 (60,3)	–	29,0/11,0***	47,3/21,9***
Stationärer Computer	38,8 (–)	41,1 (–)	68,8 (59,9)	47,6/15,9***	47,5/24,7***	78,4/44,2***
Laptop	23,6 (–)	37,3 (–)		29,0/9,6***	45,5/16,7***	
Anteil der Onliner	37,8 (47,2)	55,7 (49,8)	74,2 (58,5)	47,9/12,8***	67,6/25,4***	85,8/45,0***
*Gründe der Nichtnutzung des Internets*	**2009**	**2014**	**2019**	**2009**	**2014**	**2019**
	**Prozente (nur Offliner)**			**Prozente nach Altersgruppen** (65–79/80+)		
Es ist zu kompliziert	71,5	69,8	79,9	70,4/73,2	70,7/68,2	77,3/82,2
Sicherheitsbedenken	57,2	62,6	71,1	65,9/42,8***	67,0/56,6	77,5/67,3
Zu hoher Aufwand beim Erlernen	61,7	61,8	70,4	58,5/66,8*	63,9/58,5	65,3/73,4
Eine andere Person ruft Informationen ab	46,7	53,7	66,4	50,3/40,8**	55,6/51,0	68,0/66,2
Fehlende Unterstützung	31,9	35,7	39,1	31,1/33,3	37,0/34,0	36,0/40,5
Kosten	33,9	37,0	31,9	31,6/37,7	39,3/34,1	34,7/30,6
*Internetanwendungen*	**2009**	**2014**	**2019**	**2009**	**2014**	**2019**
	**Prozente (nur Onliner)**			**Prozente nach Altersgruppen** (65–79/80+)		
Infosuche, allgemein	81,7	86,5	93,4	83,1/69,2*	87,7/78,9*	95,1/86,0***
E‑Mails	88,5	89,9	93,8	88,8/84,5	90,9/83,3*	94,2/93,0
Fahrpläne abrufen	76,1	75,8	75,8	78,2/55,3**	76,6/68,6	79,0/61,0***
Gesundheitsthemensuche	55,1	65,7	61,3	56,4/42,1	67,3/54,3*	63,8/50,4**
Zeitungen lesen	46,3	56,5	57,5	46,6/42,1	58,8/41,1 **	60,4/44,0***
Internetbanking	38,1	40,3	54,4	39,1/28,9	43,7/18,1***	58,1/37,3***
Kauf von Waren	31,0	43,3	40,7	32,3/18,4	46,7/21,9***	44,5/22,7***
Soziale Netzwerke	5,4	16,5	28,8	5,8/2,7	17,6/9,9	30,8/19,7**

1 = Skala (1 „lehne völlig ab“ bis 5 „stimme völlig zu“); 2 = Skala (1 „trifft gar nicht zu“, 5 „trifft völlig zu“). Angezeigt sind nach Altersgruppen, Sprachregion und Bildungsgruppen gewichtete Daten. „–“  = in diesem Jahr nicht erhoben. Signifikanztests zwischen Altersgruppen: T‑Test bzw. Cramers V:

***p < 0,001, ** *p* < 0,01, *p < 0,05

## Ergebnisse

### Statistische Analysen

Neben deskriptiven Gegenüberstellungen der Verteilungen pro Erhebungsjahr wurden Vergleiche zwischen jüngere Altersgruppen (65 bis 79 Jahre) und älteren Altersgruppen (ab 80 Jahre) berücksichtigt (Tab. 2). Zusätzlich wurde eine binär logistische Regressionsanalyse pro Jahr gerechnet. Fehlende Werte wurden listenweise eingeschlossen.

### Technikinteresse und Einstellung zum Internet

Wie anhand von Tab. 2 zu sehen ist, ergibt sich 2009 bei der ersten Aussage zur Techniknutzung „Der technische Fortschritt muss immer weitergehen“ ein Mittelwert von 3,85 auf der Skala von 1 „lehne völlig ab“ bis 5 „stimme völlig zu“. Im Jahr 2014 (M: 3,82) und 2019 (M: 3,71) hat sich dieser Mittelwert nicht maßgeblich verändert. Auch die beiden weiteren Aussagen („Ohne technische Geräte könnte ich mir mein Leben nicht mehr vorstellen“ und „Ich interessiere mich sehr für neue technische Dinge“) wurden über alle Jahre hinweg ähnlich hoch bewertet, wenn auch die Aussage zum Interesse an neuen technischen Dingen etwas zurückhaltender im Vergleich zu den beiden ersten Aussagen bewertet wurde.

Neben den allgemeinen Einstellungen zu technischen Dingen konnte auch die Einstellung speziell zum Internet als eine spezifische Anwendung abgefragt werden. Es zeigt sich hier, dass auch die 3 Einstellungsfragen zum Internet (Tab. 2) über die Jahre hinweg hoch bewertet wurden, so ergibt sich z. B. bei der ersten dazugehörigen Aussage („Das Internet erleichtert den Kontakt zu anderen Menschen“) bei der Erhebung 2009 ein Mittelwert von 3,18, 2014 ein Mittelwert von 3,21 und 2019 ein Mittelwert von 3,28, auf der Skala von 1 „trifft gar nicht zu“ bis 5 „trifft völlig zu“. Innerhalb der 3 Stichproben ist erkennbar, dass sich die Einstellungsfragen zum Internet hinsichtlich der Altersgruppen (65 bis 79 Jahre und 80 Jahre und älter) statistisch signifikant unterscheiden: Ältere Personen sind dem Internet gegenüber etwas negativer eingestellt als jüngere Personen.

### Nutzung von Alltagstechnologien

96,7 % der 2009 befragten Personen gaben an, einen Fernseher zu besitzen; davon erklärten 89,0 %, dass sie diesen täglich nutzen (Tab. 2). 2014 und 2019 stieg diese Zahl nicht weiter an. Eine ähnlich stabile, wenn auch leicht rückgängige Entwicklung ist bei der Nutzung von Radio und Festnetztelefon zu beobachten. Anders sieht dies bei den klassischen Mobiltelefonen aus, die mehr und mehr durch Smartphones ersetzt werden. Gerade die Verwendung von Smartphone und Tablet ist seit 2014 stark angestiegen und belief sich 2019 auf 63,7 % bzw. 40,0 % (Tab. 2). Dennoch ist bei diesen neueren Mobilgeräten auch 2019 noch eine Differenz zwischen jüngeren und älteren Personen erkennbar: So besitzen zwar 75,2 % der 65- bis 79-Jährigen ein Smartphone und 47,3 % ein Tablet, aber nur 34,6 % bzw. 21,9 % der mindestens 80-Jährigen.

### Nutzung des Internets

Innerhalb der Erhebung 2009 nutzten 37,8 % das Internet, 2019 waren es bereits 74,2 %. Dennoch zeigte sich auch 2019 weiterhin ein Unterschied zwischen den jüngeren (65 bis 79 Jahre) und älteren Altersgruppen (80+): So nutzten 2019 zwar 85,8 % der „jüngeren Älteren“ das Internet, aber nur 45,0 % der mindestens 80-Jährigen. Es zeigt sich auch, dass nicht alle Onliner (Personen, die das Internet nutzen) täglich im Netz unterwegs sind; 2019 waren es 58,5 % (Tab. 2).

Die Nutzungsvielfalt der Onliner zeigt sich insbesondere in der aktuellen Erhebung. So werden zwar v. a. weiterhin die klassischen Anwendungen wie z. B. die Informationssuche, das Schreiben von E‑Mails und das Abrufen von Fahrplänen intensiv genutzt, aber immer mehr auch andere Anwendungen wie das Internetbanking oder das Onlinelesen von Zeitungen. Dennoch griff auch Ende 2019 nur weniger als die Hälfte der Personen auf das Internet zurück, wenn sie z. B. etwas kaufen wollten. Dasselbe galt für den Besuch sozialer Netzwerke (Tab. 2).

In den Interviews wurden die Offliner (Personen, die das Internet nicht nutzen) auch nach ihren Gründen für die Nichtnutzung des Internets gefragt. Die in allen 3 Erhebungen konstant hoch bewerteten Gründe hierfür sind Kompliziertheit, Sicherheitsbedenken und der Aufwand. Die Kosten fallen weniger ins Gewicht. Zudem wird das Internet oft auch deswegen nicht genutzt, weil jemand anders für die ältere Person Informationen aus dem Internet abruft. Dies ist ein Grund, der über die Jahre hinweg sogar noch an Bedeutung gewonnen hat. Zwischen den Altersgruppen zeigen sich bei den beiden letzten Erhebungen keine statistisch signifikanten Unterschiede.

### Einflussfaktoren der Internetnutzung

Um herauszuarbeiten, inwieweit soziodemografische Merkmale, die subjektive Gesundheit und das Technikinteresse (Gesamtskala aus den 3 in Tab. 2 vorgestellten Aussagen zur Technikeinstellung (1.1–1.3)) die Internetnutzung in den 3 Erhebungen beeinflusst haben, wurden 3 binär logistische Regressionen gerechnet (Zusatzmaterial online: Tab. 1). 2009 waren das Alter, die Bildung, das Einkommen und das Technikinteresse statistisch signifikant. Im Jahr 2014 waren es neben dem Alter, der Bildung, dem Einkommen und dem Technikinteresse auch das Geschlecht und die subjektive Gesundheit. Bei der aktuellen Erhebung 2019 waren dies ebenfalls das Alter, die Bildung, das Einkommen und das Technikinteresse. Personen, die mindestens 80 Jahre alt sind, gehören demnach seltener zu den Onlinern. Das Gleiche gilt für Personen mit einem geringeren Bildungsstatus und geringeren Einkommen. Personen mit einem höheren Technikinteresse gehören dafür eher zur Gruppe der Onliner als zur Gruppe der Offliner. Über alle 3 Erhebungen hinweg zeigen sich das Alter, die Bildung, das Einkommen und die Einstellung zur Technik als konstante Erklärungsfaktoren für die Internetnutzung. Hingegen zeigte sich 2019 kein Effekt beim Geschlecht oder der subjektiven Gesundheit.

## Diskussion

Der Vergleich der 3 Bevölkerungsbefragungen von 2009, 2014 und 2019 zeigt auf, dass die digitale Transformation, die sich im Alltag in einer Zunahme der Anwendung digitaler Technologien widerspiegelt, in allen Haushalten stattfindet – so auch in jenen der mindestens 65-Jährigen. Wurden 2009 oder 2014 nur wenige neue mobile Technologien wie das Smartphone genutzt, so waren es 2019 bereits mehr als die Hälfte, die ein Smartphone nutzten. Auch die Internetnutzung ist seit 2009 deutlich angestiegen und lag 2019 bei 74 %. Die Nutzung erklärte sich auch 2019 durch das Alter, die Bildung, das Einkommen und das Technikinteresse; Faktoren, die bereits 2009 herausgestellt wurden und auch in anderen Studien für Deutschland [9] und Europa [6] zu beobachten sind. Ferner ließen sich 2019 ähnliche Gründe für die Nichtnutzung des Internets erkennen, und auch heute werden noch nicht alle gängigen Onlineservices (z. B. Internetbanking, Kauf von Waren oder soziale Netzwerke) genutzt. Hinsichtlich des Technikinteresses und der Einstellung gegenüber dem Internet lässt auch die aktuelle Erhebung erkennen, dass zwar die Einstellungen über die Jahre eher stabil geblieben sind, aber auch jetzt noch diverse Einstellungsgruppen zu finden sind. Neben jenen mit einer positiven Einstellung gibt es auch weiterhin jene mit einer ambivalenten bis negativ geprägten Einstellung zum Internet.

Über alle 3 Befragungen hinweg zeigen sich in der multivariaten Analyse das Alter, die Bildung, das Einkommen und die Einstellung zur Technik als konstante Erklärungsfaktoren für die Internetnutzung. Somit ergeben sich auch Ende 2019 noch Unterschiede zwischen Personen im Alter von 65 bis 79 Jahren zu Personen ab 80 Jahren; auch sind individuelle Ausstattungen wie das Einkommen oder die Bildung weiterhin Ressourcen für eine Internetnutzung [6]. Hingegen zeigte sich 2019 kein Effekt beim Geschlecht oder der subjektiven Gesundheit mehr, was andeutet, dass ehemals erklärende Faktoren an Bedeutung verlieren, da heutzutage das Internet von mehr Bevölkerungsgruppen genutzt wird. Dennoch sollten gesundheitliche Aspekte bei der Internetnutzung weiterhin berücksichtigt werden, da z. B. Einschränkungen des Sehens oder Hörens direkten Einfluss auf die vollumgängliche Nutzung vom Internet nehmen können.

Auch wenn Nutzungszuwächse erkennbar sind, zeigt der Jahresvergleich auch, dass sich der digitale Graben nun v. a. innerhalb der älteren Bevölkerungsgruppe zeigt. So sind es insbesondere Personen ab 80 Jahren, die das Internet weniger nutzen und auch seltener ein Smartphone oder Tablet besitzen. Diese Nutzungsunterschiede lassen sich auch in der stationären Alterspflege erkennen, wo die Bewohnerinnen und Bewohner seltener technische Neuerungen nutzen als jüngere Personen [11, 15]. Insbesondere jene, die weniger bis gar nicht in der heutigen digitalen Welt vertreten sind, könnten sich sozial exkludiert fühlen, wenn klassische Zugänge wie ein „bedienter Postschalter“ aufgrund von z. B. Sparmaßnahmen wegfallen oder wenn – wie jetzt – pandemiebedingte fehlende direkte Kontakte nicht durch digitale Lösungen kompensiert werden können [16]. Es besteht die Gefahr, dass sich diese älteren Menschen einem „Diktat des Digitalen“ [8] gegenübergestellt sehen, wenn eine Teilhabe in der heutigen Gesellschaft bedeutet, bestimmte Technologien nutzen zu müssen.

Der Vergleich der 3 Untersuchungen hinsichtlich der Verteilung von „klassischen“ Technologien (z. B. Fernsehen und Radio) zu „modernen“ Technologien (z. B. Smartphone und Tablet) zeigt zwar schon, dass immer mehr auch neuere Informations- und Kommunikationswege genutzt werden, aber dennoch die Nutzung des Fernsehers und des Radios relativ stabil geblieben ist. Somit kann eher vermutet werden, dass die neueren IKT-Geräte nicht unmittelbar „klassische“ Zugänge ersetzen, sondern ergänzen.

Werden die vorliegenden Daten mit anderen internationalen Studien verglichen, zeigen sich ähnliche Trends in der Zunahme der „modernen“ Technologien (z. B. Smartphone, Internet, Tablet), dennoch zeigt der Vergleich mit europäischen Daten [5, 6] anhand der Internetnutzung, dass die Schweiz im Vergleich z. B. zu Deutschland insgesamt höhere Nutzungszahlen bei den Personen ab 65 Jahren aufweist. Daher sollten länderspezifische Unterschiede in der Nutzung von IKT berücksichtigt werden und die hier vorliegenden Erkenntnisse nicht 1:1 auf andere Länder übertragen werden.

Auch wenn der Zeitvergleich einen eindeutigen Trend zu „mehr Technik“ zeigt, sollte daraus nicht geschlossen werden, dass der digitale Graben in 10 bzw. 20 Jahren komplett verschwindet. Sicherlich wird sich die Nutzung der heute etablierten Alltagstechnologien ähnlich entwickeln wie die des Internets, dennoch ist davon auszugehen, dass in ca. 10 bis 20 Jahren andere Technologien den Alltag mitbestimmen werden (z. B. „virtual“ oder „augmented reality“, künstliche Intelligenz). Daher sollte innerhalb der Gerontologie nicht mehr nur gefragt werden, „wann“ der digitale Graben verschwindet, sondern „wie“ sich dieser Graben in den nächsten Jahren verschieben bzw. neu gestalten wird.

Die Gruppe der Offliner ist in ihren Merkmalen und Einstellungen heterogen, weshalb Maßnahmen immer zielgruppenbezogene oder, noch besser, individuell angepasste Lösungen berücksichtigen sollten [13]. Das Aufzeigen eines möglichen persönlichen Vorteils bei der Internetnutzung kann als anziehender Faktor dienen. Neben klassischen Schulungsangeboten sollte auch das soziale Umfeld (z. B. Familie, Freunde, Nachbarschaft) als Ressource herangezogen werden – als Impulsgeber und informelle Unterstützung [4]. Neben der reinen Vermittlung der Mediennutzungskompetenz sollte es aber auch um eine Medienbildung gehen, also die Fähigkeit, moderne Technologien nicht nur zu nutzen, sondern reflektiert zu nutzen (z. B. zu wissen, wo im Internet Gefahren lauern könnten).

### Limitationen

Bei den durchgeführten Studien handelt es sich um Querschnittsuntersuchungen; Veränderungen innerhalb einer Person können daher nicht abgebildet werden. Für die weitere Forschung wäre es daher wünschenswert, einerseits individuelle Daten im Längsschnitt zu erheben, anhand derer die intraindividuelle Einstellungs- und Nutzungsveränderung beobachtet werden könnte. Die hier vorgelegten Daten beziehen sich nur auf die Schweiz, daher sind direkte Übertragungen der Ergebnisse auf andere Länder nur für vergleichende Zwecke heranzuziehen; allfällige kulturelle Unterschiede sollten berücksichtigt werden. Zudem wurden in den 3 Befragungen Personen in Privathaushalten berücksichtigt; jedoch fehlt es an Studien zur IKT-Nutzung in den stationären Einrichtungen der Alterspflege.

## Fazit für die Praxis

- Auch wenn die Internetnutzung und Nutzung mobiler Endgeräte wie Smartphone und Tablet in den letzten 10 Jahren mehr und mehr in den Haushalten der älteren Menschen angekommen ist, bestehen weiterhin Nutzungsdifferenzen.
- Der digitale Graben verschiebt sich innerhalb der Altersgruppe der über 65-Jährigen, sodass Personen ab 80 Jahren weniger häufig z. B. das Internet nutzen als „jüngere Ältere“.
- Die Zivilgesellschaft sollte für Schwierigkeiten älterer Menschen im Umgang mit Technik sensibilisiert sein, damit Offliner nicht aus dem technikdominierten Alltag ausgeschlossen werden.

## Supplementary Information




