# Supplementary material for: Digital transformation in the households of older people
Source: Z Gerontol Geriatr. 2021 Apr 28;55(4):305–11. [Article in German] doi: 10.1007/s00391-021-01897-5 (PMC8080083; doi:10.1007/s00391-021-01897-5)
Supplement: Supplementary file 1 [file 391_2021_1897_MOESM1_ESM.docx]

Supplement Tabelle 1

| **Tabelle 1 Multivariate logistische Regression. Abhängige Variable „Internetnutzung“** | | | |
| --- | --- | --- | --- |
| **Prädiktoren** | **2009** | **2014** | **2019** |
|  | *OR* | *OR* | *OR* |
| Altersgruppe 65–79 Jahre (ref. 80+) | 6.387*** | 7.167*** | 7.560*** |
| Frau (ref. Mann) | 0.945 | 0.556** | 0.805 |
| Tertiärstufe (ref. Primar- bis Sekundarstufe II) | 3.830*** | 2.182** | 4.722*** |
| Haushaltseinkommen über CHF 4.000 (ref. unter CHF 4.000) | 2.963*** | 2.144*** | 2.201** |
| Allein lebend (ref. nicht allein lebend) | 1.060 | 0.832 | 1.118 |
| Subjektive Gesundheit (gut/sehr gut) (ref. schlechter) | 1.325 | 1.827** | 1.222 |
| Technikinteresse ^1^ | 2.256*** | 2.131*** | 2.256*** |
| ***Chi^2^/df/p*** | 309.486 / 7 / <.000 | 298.979 / 7 / <.000 | 244.982 / 7 / <.000 |
| ***Nagelkerges R^2^*** | 0.401 | 0.407 | 0.392 |
| ***N (gültige)*** | 866 | 845 | 909 |
| Abhängige Variable: Internetnutzung (1 „Onliner“/0 „Offliner“). Siehe Tabelle 1 für Skalen der unabhängigen Variablen. 1 = Gesamtskala aus den drei Aussagen (1.1, 1.2, 1.3) zur Technikeinstellung aus Tabelle 2 (1 „lehne völlig ab“ bis 5 „stimme völlig zu“). Logistische Regression (Methode: Einschluss; OR = Odds Ratio bzw. Exp(B)). Signifikanzniveaus: ***p<0,001, ** p<0,01, *p<0,05 | | | |
